# Incentivizing Rural Work Preferences Among Specialist Physicians: Protocol for a Discrete Choice Experiment

**DOI:** 10.2196/59621

**Published:** 2024-12-09

**Authors:** Anushree Joshi, Jallavi Panchamia, Apurvakumar Pandya

**Affiliations:** 1 Department of Health Policy, Management and Behavioral Science Indian Institute of Public Health Gandhinagar Gandhinagar, Gujarat India

**Keywords:** discrete choice experiment, specialist physicians, community health centers, rural retention, policy interventions

## Abstract

**Background:**

Retaining specialist physicians in rural parts of India poses a fundamental challenge, which affects the health care system’s functionality and provision of standard health care services. There has been an acute shortfall of specialist physicians in the fields of medicine, pediatrics, obstetrics and gynecology, and surgery at rural community health centers. This necessitates urgent policy focus to address the shortages and design effective rural retention strategies. In this study, which uses a discrete choice experiment (DCE), individuals choose from multiple-choice preferences that resemble hypothetical job descriptions.

**Objective:**

DCEs are a quantitative approach to assessing several aspects of job selection. This study aims to develop a detailed plan of a DCE method used to determine specialist physicians’ job choices. This protocol outlines the DCE method, which uses an exploratory sequential mixed methods research design to understand specialist physicians’ preferences and design reward packages that would effectively motivate them to work in underserved regions.

**Methods:**

The qualitative phase of the study involved identifying job attributes and their corresponding levels for the DCE. We followed a meticulous process, which included reviewing relevant literature, performing qualitative pilot work, conducting in-depth individual interviews, and consulting with medical and health experts. The quantitative phase involved generating a D-efficient orthogonal fractional factorial design using Ngene software to create choice scenarios using the identified job factors and their corresponding levels. The generated choice scenarios were blocked into 6 versions in 6 blocks. The DCE was undertaken among final-year postgraduate medical residents and specialist physicians from several health care facilities in Rajasthan. Various statistical models will be applied to explore the response variability and quantify the trade-offs that participants are willing to make for nonmonetary features as a substitute for adjustments in the monetary attribute.

**Results:**

After the ethics committee’s approval of the study, the qualitative data collection phase occurred from September to December 2021, while the quantitative phase took place from May to August 2022. Six attributes and 14 levels were identified and established through qualitative surveys. The experimental design resulted in 36 choice situations, which were grouped into 6 blocks. The preliminary investigation demonstrated that the instrument was valid and reliable. Statistical data analysis has been initiated, and the principal findings are expected to be disseminated in January 2025.

**Conclusions:**

The protocol provides a systematic framework to assess specialist physicians’ preferences regarding working in rural health care centers. This research has the potential to substantially influence the future of rural health care by laying the foundation for understanding specialist physicians’ choices, which will help design future incentive schemes, policy interventions, and research.

**International Registered Report Identifier (IRRID):**

DERR1-10.2196/59621

## Introduction

### Background

Several low- and middle-income countries (LMICs) experience massive deficits and uneven distribution of health care workers. This matter has been additionally exacerbated by the collapse of health care systems in LMICs and the global policy climate [1]. To attain the objective of universal health coverage, the supply of health care workers should be improved, alongside an effort to improve their distribution, accessibility, performance, and productivity [2]. However, such attempts are severely compromised by a lack of health care professionals motivated to practice in rural locations in LMICs [2]. To achieve the objective of universal health coverage in 2030, a total of 54 million health care workers are still needed, based on the recommendation by the World Health Organization of having 44.5 health care workers per 10,000 people [2]. According to the World Health Organization estimate, there was a global shortage of 17.4 million health care workers in 2013, of whom 2.6 million were physicians. Achieving the United Nations’ Sustainable Development Goal 3 of ensuring good health and well-being for all necessitates addressing the addressing the significant financial and policy implications of the dearth of health care professionals [2], particularly specialist physicians, in rural regions [1].

### Rising Attrition of Specialist Physicians in the Rural Public Health Sector: Indian Context

The availability of human resources (HR) is among the top crucial requirements for the effective operation of rural health services [3]. In India, the majority of health professionals are located in metropolitan areas, resulting in a severe scarcity of health care providers for the rural population [4]. Factors such as poor governance and underinvestment in the public health system in rural areas drive health workers to migrate to urban areas in search of better salary structures, good living conditions, proper working environments, better accessibility to use equipment and educational facilities for children, and greater transparency in HR policies [4-9]. International outmigration of health professionals also dramatically impacts the domestic health workforce’s availability [10]. Prior research has demonstrated that physicians raised and educated in cities were unwilling to take rural postings. The primary reasons attributed to their choice (an unwillingness to work in rural areas) were the limited scope of occupational growth opportunities, a lack of accountability, issues with access to the necessary medical resources needed to perform their jobs adequately, and the absence of appropriate work policies in the health system [11]. To improve the retention rates of specialist physicians in the rural health system, it is necessary to implement strategies that involve regular rotations between rural and urban job placements [11]. In addition, other factors such as intermittent electricity, poor road connectivity, an unreliable public transport system, and inadequate educational facilities also discourage specialist physicians from relocating to rural and remote regions, which is evident from the substantial number of vacant jobs [1,12-16]. A recent study revealed that specialist physicians were willing to relocate from rural community health centers (CHCs) to towns, cities, or district headquarters to live with their families [17].

### Specialist Physicians in the Rural Health Care Sector: Status as of March 31, 2022

In the Indian public health care sector, many sanctioned positions remain vacant, especially for specialist physicians at rural CHCs [3,4]. The country is grappling with a shortage of specialist physicians in rural CHCs, estimated at 79.5% according to the Rural Health Statistics Report 2021-2022 [3]. The Ministry of Health and Family Welfare has been facing persistent challenges in ensuring the availability of specialist physicians at most of the rural CHCs across the country. As of March 31, 2022, only 541 out of India’s 5480 operational rural CHCs had all 4 specialist physicians [3]. Throughout the past few decades, the Ministry of Health and Family Welfare and the Department of Health and Family Welfare (at the state level) have used a range of measures to retain staff in different Indian states [11]. These retention strategies have included mandatory rural postings, linking postgraduate program enrollment to rural positions, and offering specific financial incentives [11]. However, there is still a lack of information on acquiring a more comprehensive understanding of the evidence-based strategies required to resolve this issue further and design potential solutions.

The statistics on the vacancies of specialist physicians at rural CHCs are presented in Table 1.

The specialist physicians’ shortage profoundly impacts the distribution of rural health care services in the nation [3,4]. Their limited availability and uneven allocation in rural CHCs can lead to higher rates of newborn and maternal fatalities, contributing to a more significant burden of disease [17]. Thus, addressing vacancies in the public sector necessitates immediate policy focus [4] to guide essential policy measures to enhance the delivery and administration of health care services in rural areas.

**Table 1:** Vacancies of specialist physicians at rural community health centers (CHCs; N=10,000)^a^.

Area of specialty	Nationwide vacancies at rural CHCs, n (%)	Vacancies as per infrastructural requirements at rural CHCs, n (%)
Medicine	6750 (67.5)	7910 (79.1)
Surgery	7190 (71.9)	8320 (83.2)
Pediatrics	6970 (69.7)	8160 (81.6)
Obstetrics and gynecology	6300 (63)	7420 (74.2)

^a^Source: Rural Health Statistics Report 2021-2022 [3].

### The Use of Discrete Choice Experiments in Health Workforce Research

Discrete choice experiments (DCEs) were first used in market research, transportation, and environmental economics and later applied to investigate outcomes of interest in health care [18-22]. The DCE methodology identifies health workers’ preferences for a job based on its attributes. It is valuable for policy makers seeking to examine the most effective combinations of incentives or policy choices to promote health workers’ employment in underserved regions, particularly rural regions [9,23,24].

This protocol provides a detailed research method for a DCE, which will be used to quantify specialist physicians’ preferences for rural job incentives in Rajasthan. Rajasthan is among India’s largest states, considering its geography and cultural diversity [25], and it is currently facing a massive shortage of specialist physicians at rural CHCs [3].

### Research Objectives

The study will investigate the motivational job attributes valued by final-year postgraduate residents, who exhibit a higher propensity to quit their occupations and stay for shorter durations in rural areas, and specialist physicians, given the huge number of vacancies in the public health system. This will help policy makers understand the behavioral choices influencing and shaping specialist physicians’ decisions to accept rural postings. The study will specifically address the following research objectives:

1. To identify the significant job attributes valued for working in rural CHCs in Rajasthan
2. To identify the number of monetary incentives or job attributes traded off for other nonmonetary incentives or job attributes for working in rural CHCs in Rajasthan through willingness-to-pay (WTP) analysis
3. To develop incentive packages to increase the attraction and retention of specialist physicians in rural CHCs in Rajasthan as part of a retention strategy

## Methods

### Research Design

**Figure 1:**
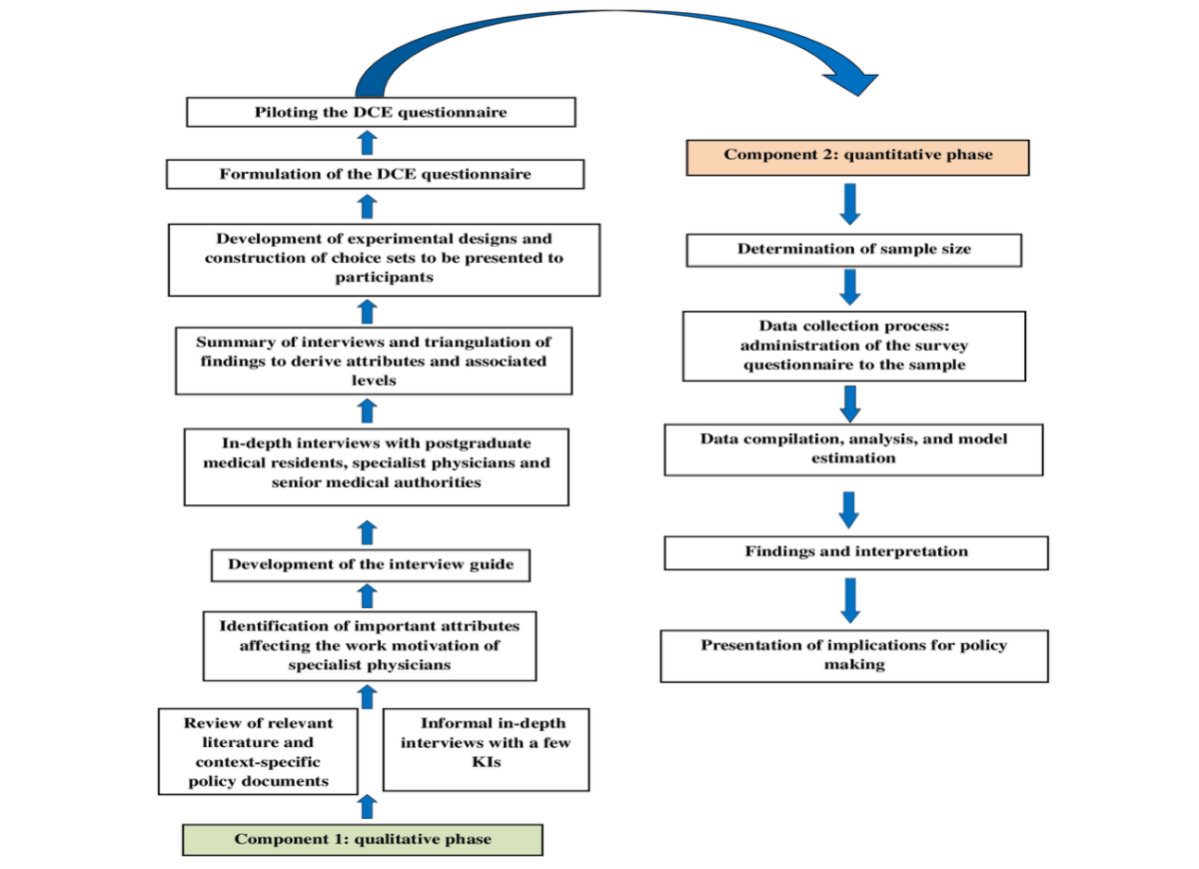
Summary of the discrete choice experiment (DCE) research design. KI: key informant.

The study research method used an exploratory sequential (qualitative+quantitative) mixed methods research design [26] to assess the incentive preferences and motivations of final-year postgraduate medical residents and specialist physicians to work in rural CHCs in Rajasthan. The survey tool for the study was constructed in 2 phases: qualitative and quantitative. The research design for the study protocol is provided in Figure 1.

### Overview of DCEs

DCEs are a precise and quantifiable method for gathering choices [9,27]. They are rooted in the theory formulated by Lancaster [28], which posits that commodities and services may be characterized by their fundamental properties, and the merit they hold for an individual is decided by a blend of these features. This approach involves providing participants with various choice profiles resembling hypothetical job alternatives and asking them to make selections. Every profile has multiple attributes that describe the job alternatives, and each attribute has multiple potential levels. Job descriptions are commonly merged to form decision sets, where participants are directed to pick the most appealing work profile. The analysis of participants’ selections among several alternatives allows for determining the relative significance of these attributes [22].

### DCE and the Theoretical Framework

The DCE method is theoretically based on random utility theory, where individuals are reasonable decision makers with perfect discrimination ability to maximize utility relative to their choices [9,24,29].

The equation presented after this paragraph shows how the perceived value of an option (*i*) in a set of choices (*C_n_*) for an individual (*n*) is broken down into 2 parts. First, there is a systematic component, which depends on the attributes of the options *V* (*X_in_*, *β*). Second, there is a random component (*ε_in_*), representing variations in preference that are not accounted for. This approach facilitates the incorporation of variations in desires across individuals, which can impact their decision-making.

*U_in_* = *V* (*X_in_*, *β*) + *ε_in_*
**(1)**

In the DCE method, it is assumed that an individual (*n*) will pick option (*i*) only if it provides the highest utility compared to all other options in the set (*C_n_*). This logical decision-making process ensures that the chosen option truly offers the most value to the individual:




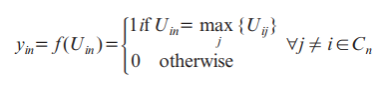

**(2)**



where *y_in_* is a choice indicator equal to 1 if alternative *i* is chosen and 0 otherwise.

### Study Settings

Rajasthan, located in India’s western region, is one of the country’s largest states in terms of geographic area. It spans 342,239 square kilometers, accounting for 10.4% of the country’s total land area [25]. It is home to 68.6 million people, representing 5.7% of India’s population. The state comprises 33 districts and 44,672 villages, with 75.13% of the population residing in villages [25,30]. Rajasthan faces an HR scarcity, with a shortage of 1939 specialist physicians for the 616 functioning CHCs [3]. In 2019, Rajasthan’s health system had only 22.02% (25,056/113,742) of the required physicians (both general and specialist) [31]. Hence, the scarcity of specialist physicians, particularly in the rural regions of Rajasthan, is a significant issue that hampers the efficient provision of health care services in such areas [32].

### Development of the DCE

To develop the DCE for the study, published recommendations in the literature were followed, including identifying criteria for acceptable research methodologies [33-36], implementing the suggestions on constructing the study’s design [36-39], and adhering to guidelines on the appropriate statistical methods [40].

### Phase 1: Qualitative Phase

One crucial aspect of building a DCE is determining the subject matter’s pertinent qualities. Attributes are usually identified by collecting primary and secondary data to customize the DCE for the specific study environment [34].

#### Identification of Job Attributes and Allocation of Associated Levels

To guide the selection of job attributes and their associated levels in a DCE, an extensive examination of existing literature and the use of qualitative methods of inquiry is advisable [23]. An attribute is a characteristic that describes a product, treatment, or decision. Levels are allocated to each attribute to define its range. Identifying attributes and their associated levels is vital for formulating job choices and designing the survey questionnaire, essentially guided by insights gained through qualitative research [23]. However, including excessive attributes and levels may overwhelm participants, impairing their decision-making abilities and potentially biasing the results. Qualitative research plays a significant role in defining attribute levels that are both pertinent and actionable for policy purposes [23,41]. Attributes and levels for DCE design were developed following the procedure outlined in recommended literature [34-39].

#### Development of the Interview Guide

To initially explore and identify key themes associated with the challenges of retaining specialist physicians in rural areas, an exploratory pilot investigation was conducted in Rajasthan to develop a semistructured interview guide [23]. The interview guide was developed in 2 phases. In the first phase, 8 key informants (KIs) were interviewed. These KIs consisted of a group of second- and third-year postgraduate medical residents, specialist physicians working in rural and urban CHCs, and other medical experts. In the second phase, expert consultations and open-ended interviews were conducted with senior administrative authorities, including health officers as well as directors and joint directors of state health institutions, to check and confirm the themes and the major rural retention challenges existing in the real-life situations. This guide was also informed by a review of relevant literature on health HR and various policy documents. This inquiry helped in building out new themes and reviewing and modifying the existing themes resulting from an emergent research design. The pilot testing allowed us to refine and assess the questions’ clarity and wording, as well as the effectiveness and appropriateness of the content in the interview guide. The final interview guide (Multimedia Appendix 1) encompassed topics related to individual job circumstances and experiences, the professional demands of the workplace, factors influencing the choice between rural and metropolitan settings, and instances of both satisfaction and dissatisfaction reported by participants. The guide also contained the relevant probes needed to elicit the desired information.

#### Sampling and Raw Data Collection

Using the semistructured interview guide, 21 telephone in-depth interviews were conducted in Rajasthan to attain data saturation. The interviews involved a varied cohort comprising specialist physicians; postgraduate medical residents; specialist physicians from the fields of medicine, pediatrics, surgery, obstetrics and gynecology (OB-GYN); and senior medical authorities with extensive experience in the public health care sector spanning 25 to 30 years. A purposive sampling technique was adopted to choose participants from several districts in Rajasthan, such as Sikar, Jhalawar, Jodhpur, Bikaner, Jaipur, Bhilwara, Jhunjhunu, and Nagaur. The choice of these districts was made to include a wide variety of geographic areas and work environments to maximize the diversity of the data. Of the 21 interview participants, 15 (71%) were male, and 6 (29%) were female; furthermore, 2 (10%) were retired senior medical officials with 25 to 30 years of experience in the field, 9 (43%) were specialist physicians employed at rural CHCs, 6 (29%) were specialist physicians employed at government district hospitals, and 4 (19%) were postgraduate medical students. To ensure data quality, notes were taken during the interviews to keep track of the information. The selection of participants from these specific specialty areas was deliberate because there existed a notable scarcity of specialist physicians within the state’s public health infrastructure.

#### Data Analysis

The interviews were transcribed from recorded conversations in the native language, and transcripts were meticulously analyzed using ATLAS.ti software (version 7.5.18; ATLAS.ti Scientific Software Development GmbH) [42]. Open coding was performed to identify the major concepts and themes emerging from the in-depth interviews. The codes were grouped into discrete clusters for each transcript and constantly compared across all transcripts. Simultaneously, memos were drafted to document the participants’ descriptions, together with the feelings, interpretations, and reactions that they associated with ongoing occurrences in their present lives.

#### Establishing the List of Job Attributes and Associated Levels

An important focus of a DCE is to thoroughly examine the optimal number of attributes in the pilot study because including too many attributes and levels may lead participants to use simplistic decision-making processes. This could result in information overload, which can create a cognitive burden and lead to inaccurate assessments of trade-offs. In studies addressing labor challenges in LMICs, the number of attributes has typically ranged from 5 to 8, with 8 being the highest [43-45].

An open coding process was used to examine and organize the job attributes, revealing the following predominant themes across all interview transcripts: physical working conditions, the structure of residential quarters, HR policies in the health system, salary and related rural financial schemes, skill development training, children’s schooling and the provision of recreational activities, transportation facilities, sanctioned staff capacity at rural CHCs, and health center locations. These themes were further narrowed down into categories to generate job attributes, and the analysis of the characteristics and range of these categories resulted in the creation of the associated levels for each attribute.

To determine the attribute levels for a DCE, several authors [38,46-50] have suggested incorporating fewer levels for each attribute to minimize measurement error. On the basis of the provided information and the established norms in the research field, 2 or 3 levels for each attribute were included.

An extensive iterative process, followed by a ranking procedure, was used to generate a final list of potential job attributes and their corresponding levels as identified by the participants (refer to the Results section), with the attributes and associated levels ranked by them in ascending order of relevance. This was further examined and discussed with the KIs to strengthen the accuracy and trustworthiness of the findings.

In addition, peer debriefing sessions were conducted within the research team to validate the final list of attributes and levels. Through a process of consensus, a definitive set of attributes, along with their associated levels, were determined. Finally, the participants were again asked to express their expectations regarding the impact of each attribute on their choice of rural job preferences. These preexisting expectations were later used to assess the theoretical validity of the questionnaire in subsequent stages. The insights gathered at each stage informed the next stage, specifically regarding triangulating the data collected across different phases [49,50].

### Phase 2: Quantitative Phase

#### Overview

The quantitative phase of the study involved constructing choice sets by combining various job attributes and their corresponding levels using different experimental designs. This process required the generation of different statistical experimental designs to optimize D-efficiency, maximize level balance and orthogonality, and minimize the estimated SEs across the parameter estimates for the job attributes [23,51]. Using Ngene software (version 1.3; ChoiceMetrics), different experimental designs were constructed to create choice sets for the study [23,51].

#### Number of Choice Sets

To proceed, hypothetical employment options, consisting of various combinations of attributes and their associated levels identified as relevant, must be created and offered to participants. [13,23]. Most health workforce studies suggest using between 7 and 16 choice sets as a feasible range [34].

In this study, we administered 15 choice sets to each participant, a quantity considered to be sufficiently minimal to prevent excessive cognitive burden while still providing high statistical precision.

#### Developing an Experimental Design: Blocking and Choice Set Creation

The next phase in developing an experimental design involves determining the choice sets and applying blocking. Blocking, a technique commonly used in DCEs, entails partitioning lengthy experimental designs into groups of equal magnitude [47,51].

Initially, a comprehensive factorial design was generated, resulting in 144 potential choice scenarios (10,296 potential choice tasks) by considering various combinations of job attributes. However, due to practical constraints, presenting so many choice situations to participants was deemed impractical. Therefore, a fractional factorial design of 24 choice scenarios with different D-errors on repeated runs was created. To ensure that the statistical requirements of orthogonality, attribute-level balance, and minimal overlap were met, a simultaneous orthogonal fractional factorial design [51] comprising 36 choice scenarios was developed and optimized to minimize D-error.

Next, a blocked orthogonal fractional factorial design [51] was used to organize the 36 choice scenarios into 6 blocks. Each block contained 6 hypothetical job scenarios to be presented to participants. Subsequently, a mathematical pairing of scenarios was conducted using a pair-wise design, resulting in the development of 90 unique choice sets divided into 6 blocks, each containing 15 (17%) distinct choice sets. We used a randomization technique to randomly assign participants to 1 of the 6 blocks, with each receiving 15 choice sets to respond to. Before being incorporated into the survey tool, all choice sets underwent thorough evaluation for relevancy. Each choice task comprised an unlabeled design featuring 2 work profiles, “Job 1” and “Job 2,” without specifying whether they were rural or urban. Furthermore, a general opt-out alternative was included in the questionnaire to enhance accuracy and minimize errors in parameter estimations [23]. In addition, 2 supplementary dominance choice sets were included to serve as rationality or internal consistency checks in the final questionnaire version, where 1 alternative outperformed the other in every domain and was anticipated to be selected by a reasonable decision maker. Participants who did not pass the dominance tests were excluded from the final analysis.

#### Attribute-Level Balancing

Attribute-level balancing ensures that each level of an attribute is equally represented in the experimental design [33-36,47]. When creating choice sets, it is essential to consider how the levels of attributes are distributed. Care was taken when designing choice sets using Ngene software [51] to ensure maximum attribute-level balance through the use of the correct algorithms outlined in the software manual.

#### DCE Survey Instrument Design

Creating a comprehensive survey instrument for a DCE involves interconnected assessments centered on attribute identification and selection, experimental design construction, and decision environment considerations [35]. The DCE survey instrument aimed at presenting participants with various job scenarios across different choice sets and eliciting their preferences based on changing job characteristics. The survey introduction included the study topic, the researcher’s name and institute, the study objectives, and the intended use of the results. The survey was divided into 3 main sections:

- Section 1 presented different choice sets, each containing hypothetical job situations with varying attributes, such as workplace infrastructure, salary, staffing levels and workload at CHCs, residential facilities, workplace location, and transfer and promotion policies. Each choice set included 2 job alternatives labeled “Job 1” and “Job 2,” allowing participants to compare and choose between them.
- Section 2 featured a mix of subjective and objective questions to encourage participants to express their opinions briefly on the issues and factors influencing their expectations and decisions regarding rural postings.
- Section 3 gathered sociodemographic information about the participants, including personal details and relevant socioeconomic variables.

#### Piloting the Survey Questionnaire

In accordance with econometric guidelines [23], a pilot study was undertaken with a sample of 30 (71%; female: n=12, 40%; male: n=18, 60%) participants out of 42 recruited participants to assess their comprehension of job attributes and associated levels, as well as their level of engagement in responding to the choice tasks provided. On the basis of the feedback received, there was minimal necessity to alter the attributes, levels, or questions presented. Nevertheless, minor alterations were implemented to the demographic profile component of the questionnaire, and an additional segment was introduced to evaluate participants’ socioeconomic status. Furthermore, the quality control of the pilot questionnaire was evaluated through various validity and reliability assessment methods along with the feedback forms [47,52]. Of note, these 30 participants involved in the pilot study were not included in the final data collection sample.

#### Validity and Reliability of the Design

**Figure 2:**
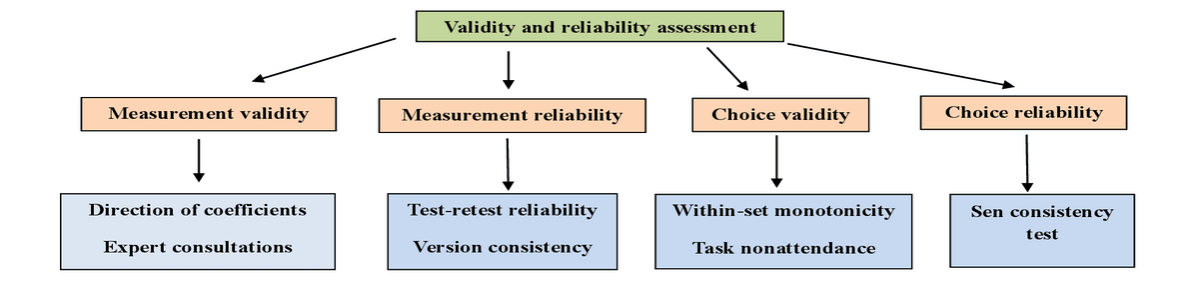
Tests for assessing the reliability and validity of the instrument.

In this study, the evaluation of the instrument design’s dependability and accuracy was comprehensive, with 7 methodologies used based on the proposed criteria [46,47] (Figure 2). These methodologies were chosen with the specific purpose of assessing the measurement validity of the design [46,47]. Content validity was initially addressed by formulating attributes and associated levels using precise qualitative techniques, such as conducting in-depth interviews. This ensured the inclusion of significant characteristics and levels for most participants. Later, during the in-depth interviews, participants were asked about their expectations regarding the influence of each level on individual preferences. This was considered a preexisting preference expectation and was analyzed to test the direction of the coefficients [46,47].

Two tests were used to check the reliability of the measurement. Test-retest reliability was used to evaluate the consistency of the participants’ responses throughout the survey, where the participant was presented with the same choice problem again, known as a repeat-choice task. The stability was evaluated based on the ratio of individuals who responded consistently across the repeat-choice tasks [53,54]. To assess the consistency of the versions, a fixed-choice problem was incorporated into the design, which was kept consistent throughout all 6 blocks.

To assess the design’s choice validity, a within-set monotonicity test was incorporated to evaluate whether participants preferred inferior levels of a characteristic over superior ones [55,56].

The second test, task nonattendance, identified individuals who consistently selected profiles from 1 specific alternative across all choice sets, considered task nonattendance [55,56].

To evaluate the reliability of the choices, we used the consistency test proposed by Sen [56], which comprises 2 principles: the expansion and contraction principles [23,55]. The expansion principle involved an initial task with 2 options (“Job 1” and “Job 2”), followed by a subsequent task with 3 options (“Job 1,” “Job 2,” and “None of the above”). The expansion feature is satisfied when a participant who selects “Job 1” does not choose “Job 2” in the subsequent challenge. The contraction principle was applied by presenting participants with an initial choice task consisting of 3 options (“Job 1,” “Job 2,” and “None of the above”), followed by a subsequent test with 2 options (“Job 1” and “Job 2”). The contraction feature is satisfied when a participant who first chose “Job 1” in the choice task also selects “Job 1” in the subsequent task. The final analysis did not include participants who were absent throughout the task and did not respond coherently to the dominant alternatives. This procedure was essential to guarantee the precision and dependability of the findings, resulting in a final pilot sample size of 30 (71%) participants out of 42 recruited participants

#### Study Population and Eligibility Criteria

Once the pilot study was completed, the proposed survey was administered to 2 groups of participants. The first group included final-year medical residents undergoing postgraduate specialty training in medicine, pediatrics, surgery, and OB-GYN at government and private medical colleges in Rajasthan and preparing to enter the health care industry. The second group consisted of specialist physicians who had been working in rural CHCs in Rajasthan for 6 to 7 years.

The selection of participants for the study considered both geographic and sex diversity. The age range of participants, spanning from >30 years to ≤50 years, was intentionally varied to encompass both young postgraduate residents and older individuals who embarked on postgraduate training later in their careers. This diverse age distribution was maintained to investigate how evolving priorities throughout the life course influence motivations to work in rural health care settings.

Participants aged >50 years were excluded from the study. In addition, we excluded health professionals from other disciplines, such as nurses, midwives, paramedical staff, laboratory technicians, and specialist physicians from other specialties, who were employed at rural health facilities within the state. Furthermore, the study did not include specialist physicians in medicine, pediatrics, surgery, and OB-GYN working at rural CHCs who had pursued Diplomate National Board certification.

#### Sample Size and Recruitment

A multistage stratified random sampling method [57] was used to recruit 156 participants from various government and private medical facilities and rural CHCs in Rajasthan, following the sample size calculations recommended for DCEs.

In research focusing on individual choices in DCEs, sample size determination typically follows the rule of thumb [35,58-63]. According to this rule, the predicted sample size is the minimum feasible quantity [58,59]. The rule of thumb proposed by Johnson and Orme [60,61] suggests that the sample size required for main effects depends on the number of choice tasks (t), the number of alternatives (a), and the number of analysis cells (c), as per the equation: N > 500 × c / (t × a). For this study, with *t*=90 (total choice tasks), a=3 (alternatives: “Job 1,” “Job 2,” and “Unable to decide”), and c=3 (the largest number of levels for any attribute), the minimum sample size was determined.

In addition, it is recommended to include at least 20 participants per questionnaire version to estimate reliable choice models [35,62,63]. Following these guidelines for DCEs [35,58-63], a minimum sample size of 120 participants was required to cover the 6 questionnaire versions.

#### Data Management and Statistical Analysis

The survey data from both participant groups were combined for quantitative analysis. The responses from the 156 participants will be assessed using Stata (version 17.0; StataCorp LLC) [64]. Digital data security measures were implemented, including encryption and restricted access.

The utility function incorporated predictor variables representing 6 job attributes, informed by the qualitative design of the DCE. Participants’ choice behavior regarding job acceptance at rural locations is the outcome variable, categorized into “Job 1” and “Job 2.” Participants’ choices will be evaluated based on various demographic and socioeconomic factors to assess their impact on decision-making.

In the DCE analysis, the probability of choice will be modeled as a function of job attributes. The effect size of each attribute will be determined by beta coefficients while controlling for other predictors at a 5% significance level. These coefficients will elucidate the individual contribution of each predictor to the model, indicating the magnitude of its impact on choice behavior when adjusted for other attribute levels and covariates.

Various statistical models (conditional logit model and fixed effects and random effects panel logit models) will be used to calculate the outcome measure. Predictor variables and covariates will be systematically entered into the regression equation based on their predicted importance.

The coefficients table for levels and covariates will be presented alongside relevant statistical measures, such as pseudo-*R*^2^, log-likelihood test, and the Akaike information criterion, to evaluate the model’s goodness of fit. Apart from the main regressions, calculations for WTP will be conducted for attribute levels. WTP quantifies the salary a participant is prepared to forgo to obtain a higher level of another job attribute, determined by the ratio between the coefficients of nonmonetary and monetary attributes [23]. This measure offers valuable insights to inform policy decisions [23,65-68].

### Ethical Considerations

The research obtained approval from the institutional ethics committee of the Indian Institute of Public Health Gandhinagar (TRC/2021-22/14). Administrative permission was granted by the Secretary of the Department of Medical, Health & Family Welfare and the Department of Medical Education, government of Rajasthan.

Due to the COVID-19 pandemic, participants recruited for the initial phase received the consent form and information sheet on the web before the qualitative interviews. They provided verbal consent for participation and recording during this phase. Telephone interviews were conducted attentively, with participants informed of their right to end the conversation at any time. For the second phase, involving the quantitative survey, participants provided written informed consent. Distinct codes were used for participant identification to maintain confidentiality. The study adhered to ethics guidelines and ensured the confidentiality of all participants.

## Results

### Overview

The collection of qualitative data occurred between September and December 2021, followed by the quantitative data collection phase conducted between May and August 2022, using diverse recruitment methods. The selected attributes (and the number of associated levels) derived from the qualitative phase of the study included workplace infrastructure (2 levels); salary, including rural retention bonus (3 levels); staffing levels and workload (2 levels); residential facilities (3 levels); workplace location (2 levels); and transfer and promotion policies (2 levels; Table 2).

**Table 2:** List of attributes and their associated levels in the discrete choice experiment.

Attributes and levels			Description
**Workplace infrastructure**			
	1	Basic infrastructure	
	2	Advanced infrastructure	
**Salary (including rural retention bonus)**			
	1	Current government salary	
	2	Current government salary+25% increase in rural retention bonus	
	3	Current government salary+50% increase in rural retention bonus	
**Staffing levels and workload**			
	1	Fully staffed CHC with moderate workload	
	2	Understaffed CHC with heavy workload	
**Residential facilities**			
	1	Provision of well-developed residential quarters, not free of charge	
	2	Provision of substandard residential quarters with limited facilities, but free of charge	
	3	No residential quarters provided but house rent allowance provided	
**Workplace location**			
	1	≥30 km from the current place of residence or hometown (30-149 km)	
	2	≥150 km from the current place of residence or hometown (150-269 km)	
**Transfer and promotion policies**			
	1	Substantial more rational policies based on seniority or genuine personal and medical needs, along with time-bound promotions	
	2	Ad hoc policies based on current practices and norms	

### Construction of Choice Sets

Using a blocked orthogonal fractional factorial design, attributes and associated levels derived from the qualitative phase of the study resulted in a construction of 90 unique choice sets partitioned into 6 blocks, each containing 15 (17%) distinct choice sets. A sample of a choice set is provided in Textbox 1.

For the final data analysis, the sample comprised 156 individuals, categorized into 4 specialist physicians’ groups: medicine (n=58, 37.2%), surgery (n=34, 21.8%), pediatrics (n=32, 20.5%), and OB-GYN (n=32, 20.5%). Statistical analysis of the data has been initiated, and the findings are expected to be disseminated in January 2025.

**Sample choice set.**

**Job 1**

- Workplace infrastructure: basic infrastructure
- Salary (including rural retention bonus): current government salary+50% increase in rural retention bonus
- Staffing levels and workload: fully staffed community health center (CHC) with moderate workload
- Residential facilities: provision of substandard residential quarters with limited facilities, but free of charge
- Workplace location: ≥150 km from the current place of residence or hometown (150-269 km)
- Transfer and promotion policies: substantial more rational policies based on seniority or genuine personal and medical needs along with time-bound promotions.

**Job 2**

- Workplace infrastructure: advanced infrastructure
- Salary (including rural retention bonus): Current government salary+25% increase in rural retention bonus
- Staffing levels and workload: fully staffed CHC with moderate workload
- Residential facilities: Provision of well-developed residential quarters, not free of charge
- Workplace location: ≥30 km or more from the current place of residence or hometown (30-149 km)
- Transfer and promotion policies: ad-hoc policies based on current practices and norms.

**Which of these 2 jobs do you prefer?**

- Job 1
- Job 2
- Unable to decide

### Results of the Validity and Reliability Assessment

A preliminary investigation was performed using a conditional logit regression model, as mentioned in the existing literature on model specification [33,69]. The analysis was performed using Stata software (version 17.0) [64]. The results were analyzed using the beta coefficients and their related 95% CIs. A significance level of 5% was used. The model demonstrated a suitable fit for DCE (pseudo-*R*^2^=0.09) [23,47]. The findings indicated that all job attributes except advanced workplace infrastructure and substandard residential facilities significantly explained the preferences for rural jobs (*P*<.05). The results aligned with the preexisting preference expectations, demonstrating the theoretical validity of the design (Multimedia Appendix 2).

The findings indicated that 71% (30/42) of the participants consistently selected repeat-choice tasks, suggesting satisfactory reliability throughout the pilot survey (Multimedia Appendix 3). It was further found that the design exhibits version consistency because there was no remarkable variation between the blocks in selecting alternatives for the fixed-choice tasks. Furthermore, the study revealed that 30 (71%) of the 42 participants made rational decisions by choosing the dominant alternative. As a result, the design’s within-set monotonicity was confirmed. The task nonattendance rate was 29% (12/42), which is within an acceptable range for a DCE. Of the 42 participants, 30 (71%) based their selections on the consistency principles proposed by Sen [56], suggesting satisfactory decision reliability. Hence, the data from 29% (12/42) of the participants who were found to have not attended to the task were excluded from the final analysis due to inadequate information. Most of the participants concurred that the design was understandable (30/42, 71%) and that the questionnaire was easy to respond to (30/42, 71%).

## Discussion

### Summary

Investing in increasing the number and accessibility of the health workforce is currently the most crucial area in India requiring policy attention [4]. The protocol presented herein seeks to bridge this knowledge gap by addressing the significant shortage of specialist physicians, a critical segment of the health care workforce, informing future policy interventions in Rajasthan, one of India’s largest states. This study uses a novel approach—DCE—to generate valuable, actionable, and policy-relevant data to improve the retention of specialist physicians in rural regions. Prior research on health HR in India has predominantly focused on general physicians, nurses, and midwives, while neglecting the importance of specialist physicians [5,7,70-72].

This study provides a thorough approach for performing a DCE and analyzes the findings of an initial survey conducted to assess the effectiveness of the study design before its complete adoption.

The pilot tests present a comprehensive overview of the essential procedures for determining job attributes and associated levels, generating choice sets, defining the utility model, selecting labeled and unlabeled choices, and implementing the design.

This study used a diverse array of methodologies to assess the efficiency of the design. Attributes were identified throughout the qualitative phase of the study through a meticulous iterative procedure involving in-depth interviews. However, the qualitative analysis revealed unique characteristics and levels specific to the location [73,74], which may add bias to the findings when examined in the broader context. With the incorporation of various attributes and associated levels, several experimental designs were created using an orthogonal fractional factorial design to create choice sets that guaranteed orthogonality, balance, and little overlap between attribute levels. Generic label options were used to create choice sets. Although specific labeling more accurately reflects individual preferences and actual choices, generic labeling is preferable for analyzing the trade-offs among multiple characteristics [38].

The direction of the coefficients validates the theoretical validity, demonstrating that the instrument effectively assesses participants’ choices [47,74] and that the attributes have been assigned appropriate levels, allowing for effective trade-offs [23]. Furthermore, the outcomes of the reliability and validity evaluations of the design indicate that it exhibits response efficiency [47].

By conducting a DCE, this study will serve as a cornerstone in providing a systematic and comprehensive understanding of several job characteristics, individual traits, and the importance of work incentives for specialist physicians in rural areas. This study’s findings will inform and shape judicious policy measures concerning implementing attractive work incentive schemes to attract and retain specialist physicians in rural areas. This will allow policy makers and health care administrators to refine their strategies for managing attrition and talent migration, thereby facilitating the development of a policy evaluation framework and planning health care HR for the state.

### Strengths and Limitations

The primary advantage of a DCE involves its experimental nature, which allows for recording participants’ preferences under controlled experimental conditions, which helps to gain insight into how attribute changes affect individuals’ choices. Second, the job attributes and their corresponding levels were produced by performing comprehensive and precise objective evaluations, enhancing the design quality and result validity. The study identifies a wide variety of job characteristics that can be modified and adjusted to suit the preferences of specialist physicians in different national and international scenarios. Furthermore, pilot research was conducted using established frameworks to develop a thorough strategy for designing and assessing the quality of the DCE instrument.

While DCEs offer benefits, their implementation presents numerous challenges. At first, the study provided participants with hypothetical work options developed using qualitative research to collect relevant attributes. By using experimentally determined hypothetical alternatives during qualitative interviews, it became feasible to examine the participants’ “expressed preferences” rather than their “revealed preferences.” Moreover, the primary constraints of DCE methodologies stem from the cognitive challenge posed by the intricate assortment of options presented in bundles that encompass numerous attributes and levels. This can present several intricate trade-off questions to optimize statistical efficiency. Furthermore, the DCE’s distinct emphasis on specialist physicians guarantees that the results are particularly relevant to this group.

### Dissemination of Study Findings

The results of this study will be shared through various platforms, such as scholarly journals that undergo peer review, conferences, and policy briefs. A concise summary of the findings will be made available to the participants on demand.

### Future Directions

Further research is necessary to fully understand the broader context in which specialist physicians operate and the influence of the sociopolitical environment on their professional independence within the rural health care system to improve the retention and job satisfaction of rural specialist physicians. It is essential and equally important to further investigate and examine the factors that influence the uneven distribution and scarcity of female specialist physicians. To optimize the allocation of scarce monetary resources and conduct thorough assessments of policy initiatives, it is necessary to consider their costs, the accuracy of cost information, funding sources, and long-term sustainability. To comprehend the expenses linked to the policy intervention, a financial assessment of all resources used in executing the intervention is necessary.

### Conclusions

This study offers a comprehensive process for building the DCE instrument and presenting the findings of instrument pilot testing. This study also demonstrated a unified and thorough framework that may be applied in future research endeavors. This study will be helpful in investigating the job factors needed to understand this issue thoroughly. Moreover, the study findings will aid in designing rural intervention packages, essential in promoting relevant policy measures to address the high turnover rates of specialist physicians in Rajasthan. In addition, it will offer valuable evidence to guide future policy interventions globally, facilitating the creation of incentive packages aimed at attracting and retaining specialist physicians in rural and underserved areas of LMICs.

## Appendix

Multimedia Appendix 1 Interview guide.

Multimedia Appendix 2 Conditional logit regression analysis (pilot survey).

Multimedia Appendix 3 Outcomes of reliability and validity assessments of the design.

## Abbreviations

CHC: community health center
DCE: discrete choice experiment
HR: human resources
KI: key informant
LMICs: low- and middle-income countries
OB-GYN: obstetrics and gynecology
WTP: willingness-to-pay

## Data Availability

The datasets generated and analyzed during this study are available from the corresponding author on reasonable request.
