# Supplementary material for: Incentivizing Rural Work Preferences Among Specialist Physicians: Protocol for a Discrete Choice Experiment
Source: JMIR Res Protoc. 2024 Dec 9;13:e59621. doi: 10.2196/59621 (PMC11667135; doi:10.2196/59621)
Supplement: Multimedia Appendix 1 [file resprot_v13i1e59621_app1.pdf]

Interview Guide

UNDERSTANDING WORK MOTIVATION AND INCENTIVE PREFERENCES TO IMPROVE RURAL RETENTION AMONG SPECIALIST PHYSICIANS IN RAJASTHAN, INDIA: A DISCRETE CHOICE EXPERIMENT

SUBMITTED BY:

ANUSHREE JOSHI

RESEARCH SCHOLAR, IIPHG

Themes	Researcher's Questions	Interviewer questions	Probes
Personal work situation & Experience	• Opening rapport building questions about the participants to know their educational & professional history.<br>• Details about their family background<br>• Sociodemographic details.<br>• Professional goals<br>• Current & previous work experience in rural /urban location if any.	• Please tell me about yourself, your background & your objective of joining the medical profession.<br>• I would like to know about your experience & journey as a medical professional so far.	• City of origin<br>• Married or single<br>• No. of years married, do you have children (If yes, how many)<br>• Is your spouse also in the medical profession?<br>• Did parental ambitions, childhood experience influence your reason for entering the medical profession or was it about your idea of service & contribution to the society?<br>• Personal background is rural or urban?<br>• Is your family also established in the same background? (urban set up or rural set up)<br>• Years of service in the current profession, Monthly household income (optional)

Themes	Researcher's questions	Interviewer questions	Probes
Professional job demands/challenges at community health centres/semi-rural regions of Rajasthan	• To understand the different kinds of challenges perceived by medical professionals , when they are asked to join community health centres / semi-rural areas.<br><br>• To understand the qualitative, quantitative & organizational job demands experienced by medical professionals at community health centres/semi-rural regions of Rajasthan.	What does it feel like to work in semi-rural regions or to be a government specialist doctor at Community Health centres (CHC) of Rajasthan?	**Quantitative demands:**<br>• Working conditions (shortage of water, electricity, space, etc)<br>• Work overload<br>• Unfavourable shift work schedule<br>• Low salaries<br>• Availability of material resources<br>• Irregular working hours<br>• Workplace location<br><br>**Qualitative demands:**<br>• Emotional demands<br>• Cognitive/mental demands<br>• Work-home conflict<br>• Harrassment by patients<br>• Threat of physical violence<br>• Demanding contacts with patients<br>• Problems of access & communication<br>• Societal politics<br>• Life security<br>• Local environment

Themes	Researcher's questions	Interviewer questions	Probes
Professional job demands/challenges at community health centres/semi-rural regions of Rajasthan	• To understand the different kinds of challenges perceived by medical professionals , when they are asked to join community health centres/semi-rural areas.<br><br>• To understand the qualitative, quantitative & organizational job demands experienced by medical professionals at community health centres/semi-rural regions of Rajasthan.	What does it feel like to work in semi-rural regions or to be a government specialist doctor at Community Health Centre (CHC) of Rajasthan?	**Organizational Job demands:**<br>• Lack of transparency related to transfer & promotion policies.<br>• Workplace infrastructure<br>• Bureaucracy<br>• Job satisfaction level<br>• Interpersonal conflicts with the colleagues<br>• Lack of cadre system<br>• Staffing level (lack of subordinate staff)

Themes	Researcher’s questions	Interviewer questions	Probes
Job Resources (Financial & Non-financial incentives)	• Understanding the extrinsic factors of work motivation for joining community health centres/semi-rural regions of Rajasthan?<br><br>• To get the understanding of financial & non-financial incentives, which can push respondents to think of working in such regions for a long period of time.	What will motivate you to join & remain in Community Health centres of Rajasthan?<br>Please elaborate in detail, with example (if possible)	**Financial Incentives:**<br>• Salary levels<br>• Rural retention bonus<br>• Other benefits (if any)<br><br>**Non-Financial incentives:**<br>**Social Resources**:<br>• Co-worker support<br>• Head/in-charge support<br>• Good education for children<br>• Co-location with spouse<br>• Transportation facilities<br>• Residential facilities

Themes	Researcher's questions	Interviewer questions	Probes
Job Resources (Financial & Non-financial incentives)	• What are the extrinsic factors of work motivation for joining community health centres/ semi-rural regions of Rajasthan?<br><br>• To get the understanding of financial & non-financial incentives, which can push respondents to think of working in such regions for a long period of time.	What will motivate you to join & remain in Community Health Centres of Rajasthan?<br>Please elaborate in detail, with example (if possible)	**Non-Financial incentives:**<br>**Work Resources**:<br>• Availability of material resources such as equipments & drugs.<br>• Job autonomy<br>• Flexible time arrangements<br>• Workplace infrastructure<br>• Skill utilization (Basic equipments for routine investigation)<br>• Management style<br><br>**Organizational Resources:**<br>• Rational procedures for transfer, promotion & placement (Procedural fairness)<br>• Participation in decision making<br>• Performance feedback<br>• Workplace culture

Themes	Researcher's questions	Interviewer questions	Probes
Job Resources (Financial & Non-financial incentives)	• What are the extrinsic factors of work motivation for joining community health centres/ semi-rural regions of Rajasthan?<br><br>• To get the understanding of financial & nonfinancial incentives, which can push respondents to think of working in such regions for a long period of time.	What will motivate you to join & remain in Community Health Centres of Rajasthan?<br>Please elaborate in detail, with example (if possible)	**Non-Financial incentives:**<br>**Provision of career development resources:**<br>• Skill development & capacity building<br>• Possibilities for learning & professional development<br>• Skill upgradation

Themes	Researcher's questions	Interviewquestions	Probes
Personal preferences for joining community health centres/semi-rural regions of Rajasthan	What are the intrinsic factors of work motivation for joining community health centres/semi-rural regions of Rajasthan?	Is there anything in you as a person, which inclines you towards this kind of profession?	• Personal values of service<br>• Personal fulfillment<br>• Familial values<br>• Disinclination for private practice<br>• Geographical affinities (upbringing, community & culture)<br>• Rural upbringing<br>• Familiarity with village life

How challenging is it for female medical professionals to work at community health centres in comparison to male medical professionals?

Is there any story, you want to share, where you felt happy & motivated as a doctor.

Also, a story, where you felt unhappy & demotivated as a doctor.

Thank You!!
