# Supplementary material for: Incentivizing Rural Work Preferences Among Specialist Physicians: Protocol for a Discrete Choice Experiment
Source: JMIR Res Protoc. 2024 Dec 9;13:e59621. doi: 10.2196/59621 (PMC11667135; doi:10.2196/59621)
Supplement: Multimedia Appendix 2 [file resprot_v13i1e59621_app2.docx]

**Pilot survey findings resulting from conditional logit regression (N=30).**

| Attributes and levels | Coefficient (95% CI) | Standard Error | *P* value |
| --- | --- | --- | --- |
| **Salary (including rural retention bonus)**  Current government salary^a^ | 0.34 (0.17- 0.51) | 0.08 | *P* < .001 |
| **Workplace infrastructure**  Basic workplace infrastructure  Advanced workplace infrastructure | Reference  0.23 (-0.05 to 0.51) | 0.14 | *P* = .11 |
| **Transfer and promotion policies**  Ad hoc policies based on current practices and norms.  Substantial more rational policies based on seniority, along with time-bound promotions. | Reference  0.66 (0.38- 0.95) | 0.14 | *P* < .001 |
| **Residential facilities**  No residential quarters, but house rent allowance provided.  Provision of well-developed residential quarters, not free of charge  Provision of sub-standard residential quarters, not free of charge | Reference  0.58 (0.22- 0.93)  0.33 (-0.02 to 0.70) | 0.18  0.18 | *P* =.002  *P* = .06 |
| **Workplace location**  >150 km from the current place of residence or hometown (150 km -269 km)  >30 km from the current place of residence or hometown (30 km -149 km) | Reference  0.62 (0.33-0.91) | 0.14 | *P* < .001 |
| **Staffing levels and workload**  Understaffed CHC with heavy workload  Fully staffed CHC with moderate workload  **Model diagnostics**  Number of Participants 30  Number of Observations 900  Log Likelihood -529.74  AIC 1075.48  BIC 1113.90  Prob > chi^2^  *P* < .001 | Reference  0.53 (0.25-0.82) | 0.14 | *P* < .001 |

a: continuous variable
