# Supplementary material for: Incentivizing Rural Work Preferences Among Specialist Physicians: Protocol for a Discrete Choice Experiment
Source: JMIR Res Protoc. 2024 Dec 9;13:e59621. doi: 10.2196/59621 (PMC11667135; doi:10.2196/59621)
Supplement: Multimedia Appendix 3 [file resprot_v13i1e59621_app3.docx]

Outcomes of the validity and reliability assessments in terms of the proportion of participants who responded to the different tests.

| Tests | Block A | Block B | Block C | Block D | Block E | Block F | Total |
| --- | --- | --- | --- | --- | --- | --- | --- |
|  | n (%) | n (%) | n (%) | n (%) | n (%) | n (%) | N (%) |
| **Measurement reliability** |  |  |  |  |  |  |  |
| Test-retest reliability | 5 (71.5) | 5 (71.5) | 5 (71.5) | 5 (71.5) | 5 (71.5) | 5 (71.5) | 30 (71.5) |
| Version consistency | 5 (71.5) | 6 (85.7) | 5 (71.5) | 4 (57.1) | 5 (71.5) | 5 (71.5) | 30 (71.5) |
| **Choice validity** |  |  |  |  |  |  |  |
| Within-set monotonicity | 5 (71.5) | 4 (57.1) | 5 (71.5) | 5 (71.5) | 6 (85.7) | 5 (71.5) | 30 (71.5) |
| Task nonattendance | 2 (28.5) | 3 (42.8) | 2 (28.5) | 2 (28.5) | 1 (14.2) | 2 (28.5) | 12 (28.5) |
| **Choice reliability** |  |  |  |  |  |  |  |
| Expansion consistency | 5 (71.5) | 4 (57.1) | 6 (85.7) | 5 (71.5) | 5 (71.5) | 5 (71.5) | 30 (71.5) |
| Contraction consistency | 6 (85.7) | 4 (57.1) | 6 (85.7) | 4 (57.1) | 5 (71.5) | 5 (71.5) | 30 (71.5) |
